# Burning of municipal waste in household furnaces and the health of their owners

**DOI:** 10.1038/s41598-024-83572-4

**Published:** 2024-12-30

**Authors:** Alicja Kicińska, Grzegorz Caba, Fernando Barria-Parra

**Affiliations:** 1 Faculty of Geology, Geophysics and Environmental Protection, Department of Environmental Protection, AGH University of Krakow, Mickiewicza 30 Av., 30-059 Kraków, Poland; 2 Prospecting and Environment Laboratory (Promediam), Universidad Politecnica de Madrid, Alenza 4, 28003 Madrid, Spain

**Keywords:** PM2.5, PM10, TVOC, HCHO, Health risk, Climate sciences, Environmental sciences

## Abstract

The aim of the study was to determine the scale of emission and airborne dispersion of selected pollutants (PM2.5, PM10, TVOC, HCHO) associated with the combustion of various types of municipal waste (MW), its mixed stream and separate fractions, in a household furnace, as compared to conventional (CF) and alternative (AF) fuels. We demonstrated that each type of fuel (AF, CF, AFw) combusted in a household furnace is a significant source of air pollutants, especially fine PM2.5 particles, whose concentrations exceeded the limit values (3.1–17.2 times for PM2.5 and 0.5–7.4 times for PM10). The combustion of MW in household furnaces generated higher levels of PM2.5 (up to 345 µg/m^3^) and PM10 (up to 369 µg/m^3^) than AF or CF, at the same time being a significant source of TVOC (up to 0.3 mg/m^3^) and HCHO (0.4 mg/m^3^). The analysis showed that according to the Polish and European classification, air quality (AQI) during the combustion of all the materials analyzed is very poor (*n* = 12) or extremely poor (*n* = 19). The combustion of such materials as polystyrene, rubber and upholstery foam in household furnaces generates drastically high health risk to local inhabitants. We found that the combustion of polystyrene generated the highest Cancer Risk (CR) values of 1.04E-01 (children) and 2.60E-02 (adults), exceeding the acceptable level multiple times (CR > 10^–6^). Inhalation exposure to very poor air quality can lead to health problems, such as disorders of the respiratory, cardiovascular and immune systems. Additional risk is posed by solid fuel combustion in rural areas, which may be a significant factor deteriorating the chemical condition of soils, especially those used for agricultural purposes.

## Introduction

Out of all contemporary environmental factors, air pollution has the greatest negative impact on human life and health^1–3^. Depending on the type and time of exposure to harmful substances in the air, people may suffer from various conditions, which can even lead to diseases, predominantly of the circulatory and respiratory systems, but also the reproductive and nervous systems^4–6^. Air pollution is most harmful to children, the elderly and the chronically ill^7,8^. According to statistical data for Poland, almost 30% of children under the age of 2 and over 50% of the population over 60 years of age receive continuous medical care^9^. It is estimated that several million people around the world die every year due to poor air quality^10–12^.

Poland is a European country where there is still much to be done with regard to air quality improvement^13–15^. Air quality in Polish cities is among the poorest in the European Union^16^. This pertains to the above-standard levels of such substances as PM10 and PM2.5 particulates and benzo(a)pyrene. In 2011, six out of ten European cities having the highest number of days with exceeded permissible daily concentration of PM10 were located in Poland^17^. Undoubtedly, this stems from the fact that fossil fuels are the main materials for the energy sector, which are used e.g., for the heating of households and public institutions ^18^. Although the data refers to a situation from over 10 years ago, air quality in Polish cities has remained poor or very poor^17^. Therefore, the country had to take corrective action in terms of reducing low emissions (anti-smog resolutions requiring furnace and coal boiler replacement), installing renewable sources of energy systems (i.e., the use of biogas, installation of photovoltaic panels, solar collectors, heat pumps), heating residential and public buildings, and expanding the district heating and gas network^19^. Measures taken to reduce transportation emissions have mainly focused on the purchase of low- or zero-emission public transportation buses, construction of new routes for public and bicycle transportation, modernization and repair of tramway tracks, creation of paid parking zones and more frequent removal of dust accumulated on the roads. Actions taken to reduce industry emissions have included inspections at particularly harmful plants, implementation of the best available techniques (BAT) and creation of an emission database based on the permits issued for the largest industrial plants. These activities are sanctioned by relevant legislation.

In terms of the air quality law, and in particular the reduction of low emissions, two European Union directives are of greatest importance. The first one is Directive 2008/50/EC of the European Parliament and of the Council of 21 May 2008 on ambient air quality and cleaner air for Europe^20^, also called the CAFE Directive (Clean Air for Europe). It is the main legal act in the European Union concerning the reduction of emissions of such pollutants as: PM10, PM2.5, O_3_, SO_2_, NO_2_, NO_x_, CO, Pb and C_6_H_6_. The second act is Directive 2004/107/EC of the European Parliament and of the Council of 15 December 2004 relating to arsenic, cadmium, mercury, nickel and polycyclic aromatic hydrocarbons (PAHs) in ambient air^21^. It concerns the reduction of PAH emissions, especially B(a)P, generated mainly in the household and municipal waste sector. It sets out the target values for As, Cd, Ni and B(a)P measured in PM10, the number of sampling points, the criteria for selecting their location, the data quality required and the reference measurement methods.

Poor air quality is largely due to PM10, PM2.5 and benzo(a)pyrene in PM10, which are mainly emitted as a result of burning low-quality fuels in households^1,22–25^. What is also observed in Poland is the practice of burning municipal waste in household furnaces^18^, although it raises many controversies and is legally prohibited by national and local law^26^. Household furnaces are mainly charged with solid fuels, i.e., hard coal, wood or wood pellets. Sadly, it is still common to encounter illegal practices of burning selected fractions of municipal waste in these furnaces. The quality and quantity of waste generated from combustion, i.e., ash, depends on the thermal decomposition technology (technological parameters of the boiler) and the type of the raw material used in the system^18,27^.

In rural areas in Poland, the proportion of municipal waste in the total waste collected is decreasing, unlike in the other European countries^28^. This decline does not result from green behavior, whose aim is to reduce waste generation, but stems from the fact that some waste fractions, or worse—their mixed stream, are burnt in household furnaces. This action is aimed at reducing the cost of heating by using the heat obtained from burning this “free furnace fuel”. According to a study by Lim et al.^24^, this practice is closely related to people’s income level and economic situation. As shown by Kicińska and Caba^29^, the toxic properties of ash are determined by its morphological and chemical composition. Compounds contained in ash are readily leached and dispersed in the water-soil environment. Excessive concentrations of these substances, i.e., sulfates, phosphates, chlorides, oxides of silicon, aluminum, magnesium, iron, and calcium, and potentially toxic elements, including: Cd, Cu, Ni, Cr, Zn etc., which are the main constituents of ash, have an adverse effect on the components of the environment, as demonstrated in studies by AlMulla et al.^30^ and Wei et al.^31^. These substances directly pollute the soil, water and air, and have an indirect effect on the entire ecosystem and the health of organisms living in it^1,32,33^.

The consumer market offers numerous classes of household furnaces (boilers) for burning various types of solid fuels, both conventional (hard coal, lignite) and alternative (coal pellets, pellets), as well as biomass (wood, green waste). However, these appliances are not suitable for burning waste, and especially not the mixed waste stream. There are various chemical reactions taking place in the combustion chamber (at a relatively low temperature of 250–480 ⁰C), which are associated with the presence of different materials (e.g., PVC, paints, adhesives, varnishes, solvents, plastics etc.). As a result, volatile compounds (e.g., formaldehyde or organic compounds) and harmful or even toxic substances are emitted through exhaust chimneys^34,35^.

In light of these facts, the main aim of the present study was to determine the emission and airborne dispersion of selected pollutants (PM10, PM2.5, HCHO – formaldehyde and TVOC – total volatile organic compounds) associated with the combustion of various types of municipal waste, its mixed stream and separate fractions, in a household boiler. The other research objective was to assess health risk associated with the effect of these pollutants on local inhabitants. An innovative element of the work is that it demonstrates the degree of harm caused by the combustion of individual fractions of municipal waste in inadequate systems, such as household furnaces.

## Research area

The air quality analysis was conducted in the village of Żydów, located in Igołomia-Wawrzeńczyce commune, in Małopolska Province (Poland, EU). Igołomia-Wawrzeńczyce is a typically rural commune, with some extent of service and manufacturing activity. Agricultural land accounts for 90.5% of the commune’s area, forests and woodlands for 0.6% and surface water for 2.8%. Housing and transportation areas take up over 5.2% of the area and the remaining area (0.9%) is wasteland. The surface area of the village is 2 ha and the number of inhabitants is 188. About 90% of inhabitants have connections to mains gas. All the residential, public, and commercial and service buildings in the commune have individual heating systems powered by coal (80%) and gas (20%). All the public buildings have gas-fired boiler rooms.

The area’s climate is a combination of subcontinental and central European climate. The average daily temperature is 8.7 °C (13.3 °C during the day and 4.1 °C at night). The annual rainfall is 672 mm^36^. The heating season lasts from October to the end of April (7 months).

**Fig. 1:**
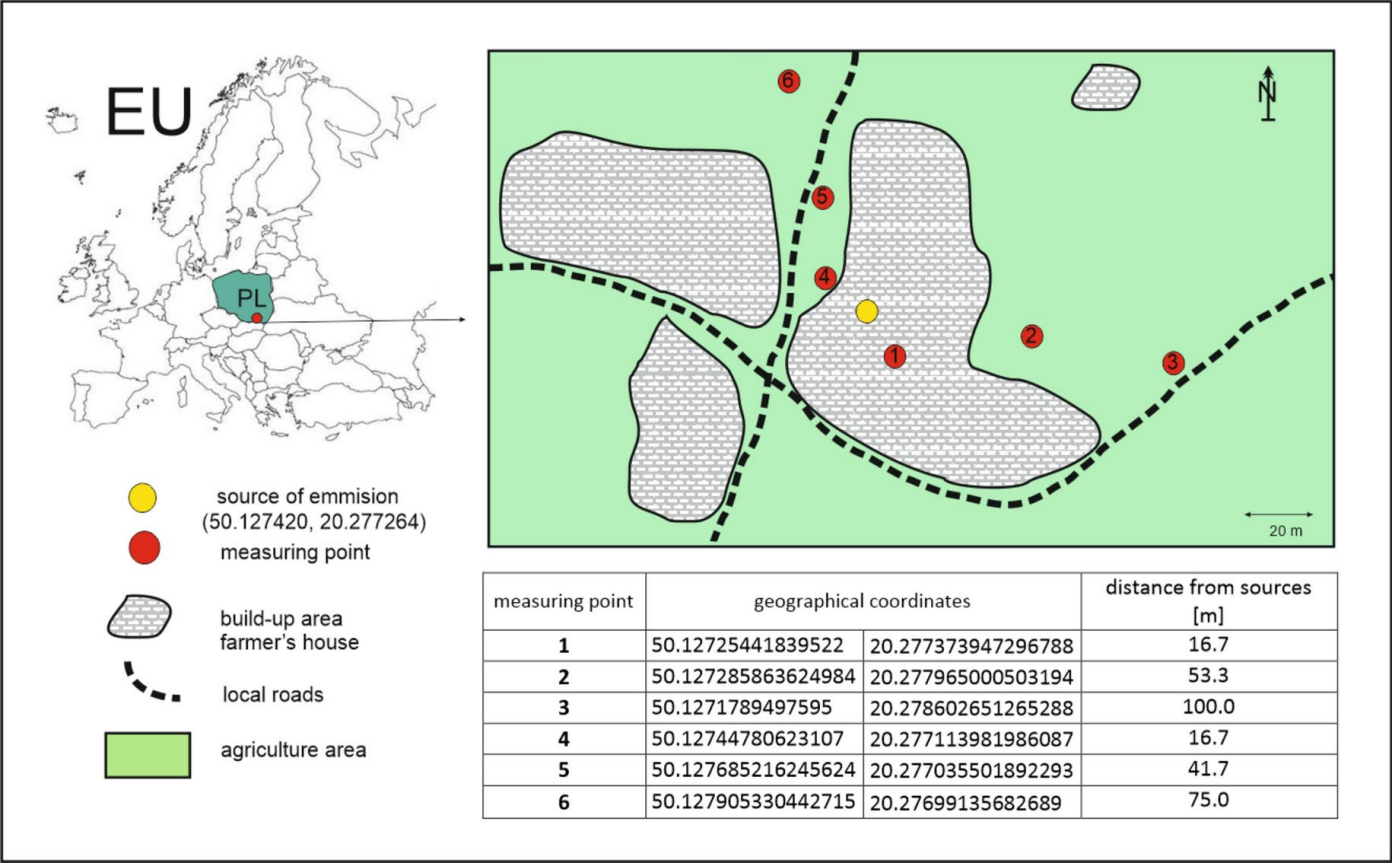
Location of research area and measured points.

The selected air quality parameters were measured at six sampling points in Żydów (Fig. 1). Their selection was determined by: site accessibility, lack of tall vegetation, and location relative to the source of emissions (mainly due to the dominant wind direction, west: points 2 and 3, south-west: point 1, and north-east: points 4, 5 and 6). The distance between the emission source where the collected material was combusted (Fig. 1) and sampling points 1–6 was as follows (data in m): 16.7, 53.3, 100, 16.7, 41.7 and 75.

## Research materials and methods

The 32 materials (Table 1) incinerated in a Keller 10 kW chamber furnace (with nominal heating capacity of 10 kW, efficiency of 80%, and maximum working pressure of 0.25 MPa) located in a private household were as follows:

- conventional fuels (CF, *n* = 3) comprising hard coal from various fuel storage sites,
- alternative fuels (AF, *n* = 8) including: coal pellets, wood pellets, straw and green waste,
- alternative fuels—wood (AF_W_, n = 5) including the wood of walnut *Juglans* L., willow *Salix* L;, acacia *Acacia* Mill. and oak *Quercus* L,
- mixed municipal waste (MMW, *n* = *3*), comprising mixed samples of all waste fractions generated in households,
- municipal waste (MW, *n* = *11*), comprising the following separately collected fractions: paper, textiles, plywood, upholstery foam, artificial leather, rubber, polystyrene and PVC packaging (used to store mineral oils or plant protection agents).

**Table 1:** List of burning materials and climatic conditions.

No	Abb	Burning material	Date	Climatic conditions				
				Wind speed [km/h]	Wind direction [−]	Pressure [hPa]	Temperature [°C]	Precipitation [mm]
1		Wood of wet willow	06-02-2022	37.25	SW	1010.75	+ 2	0
2		Wood of dry willow	05-02-2022	30.00	W	1017.75	+ 1	0
3	AFw	Wood of acacia	01-02-2022	22.75	W	1011.00	− 2	1
4		Wood of oak	03-02-2022	14.25	NW	1022.25	− 2	2
5		Wood of nut	02-02-2022	22.75	W	1003.50	− 1	0
6		Coal I	15-01-2022	21.50	W	1028.50	− 1	0
7	CF	Coal II	15-01-2022	21.50	W	1028.50	− 1	0
8		Coal III	16-01-2022	24.50	W	1020.75	− 1	0
9		Coal pellets (ekogroszek) I	16-01-2022	24.50	W	1020.75	− 1	0
10		Coal pellets (ekogroszek) II	19-01-2022	14.25	W	1026.75	− 3	0
11		Coal pellets (ekogroszek) III	20-01-2022	38.25	W	1011.00	− 2	2
12		Straw I	23-01-2022	12.75	W	1031.75	− 6	0
13	AF	Straw II	23-01-2022	12.75	W	1031.75	− 6	0
14		Wood pellets I	21-01-2022	27.25	W	1019.25	− 3	0
15		Wood pellets II	22-01-2022	22.00	NW	1031.75	− 6	0
16		Leaves of tree	23-01-2022	12.75	W	1031.75	− 6	0
17		Green waste	30-01-2022	46.00	W	1005.50	+ 1	1
18		Mixed municipal wastes I	07-01-2022	18.00	NE	1016.50	− 7	7
19		Mixed municipal wastes II	08-01-2022	9.25	S	1029.25	− 11	0
20	MMW	Mixed municipal wastes III	08-01-2022	9.25	S	1029.25	− 11	0
21		Mixed municipal wastes IV	08-01-2022	9.25	S	1029.25	− 11	0
22		PCV packaginig (mineral oil)	29-01-2022	0.00	SW	1013.38	− 2	0.3
23		PCV packaging (plant prot.)	29-01-2022	0.00	SW	1013.38	− 2	0.3
24		PCV packaging (mix oil)	29-01-2022	0.00	SW	1013.38	− 2	0.3
25		Plastic-coated paper cartons	14-01-2022	7.00	W	1022.00	− 8	0
26	MW	Polystyrene	30-01-2022	46.00	W	1005.50	+ 1	1
27		Rubber	28-01-2022	0.00	W	1025.25	− 2	4
28		Imitation leather	13-01-2022	9.00	SW	1034.00	− 8	0
29		Waste paper	06-02-2022	13.00	SE	1032.00	− 9	0
30		Sponges	29-01-2022	0.00	SW	1013.38	− 2	0.3
31		Textiles	06-02-2022	13.00	SE	1032.00	− 9	0
32		Plywood	26-01-2022	0.00	SW	1019.88	− 1	0.3

AFw, alternative fuels (wood); CF, conventional fuels; AF, alternative fuels; MMW, mixed municipal astes; MW, municipal wastes.

The self‐ignition temperatures (data in °C) were as follows: 450–550 for hard coal, 250–280 for peat, 210–320 for wood (different types), 230 for straw, 550 for coal pellets, and 180–260 for municipal waste (different types). A full description of the furnace charge preparation process has been provided in a paper by Kicińska and Caba^37^. The weight of the incinerated material, which had previously been fragmented, homogenized, reduced and divided into feed portions, was about 5 kg.

CF and AF were collected selectively (i.e., based on the type that is most commonly used in households in the study area). On the other hand, MW (dry fraction) was collected into a separate container and then processed by grinding or crushing. In this part of the study, maintaining similar combustion conditions was of utmost importance.

Statistical calculations and data presentation were conducted with the Statistic ver. *13.3* and Excel applications. The differences between means were detected by the Tukey HSD test at a significance level of *0.05*. To analyze the possible effect of the burning of different materials on air quality parameters (PM2.5, PM10, HCHO and TVOC), as well as the possible influence of atmospheric conditions (wind direction, speed of wind [km/h], pressure [hPa], temperature [°C] and rain [mm]) recorded during sampling at different sampling points, a regression analysis was performed in R^26^. The lm() function within the Stats package was used for this purpose. This function allows the analysis of the effect on continuous variables (air quality parameters) produced by categorical or factor variables (i.e. fuel type, sampling point, wind direction) and numerical variables (wind speed, atmospheric pressure, temperature and precipitation). The application of this tool allows for determining whether the changes in the average concentration of the different atmospheric pollutants associated with the value of one of the categorical variables (for example, the burning of a certain fuel or a given sampling point) are statistically significant.

The air quality parameters measured were: PM2.5 and PM10 [measured in μg/m^3^], and HCHO and TVOC [mg/m^3^]. The measurement of each parameter was repeated twice and the result included in the analysis was the average value. The measurements were performed at six points (1–6, Fig. 1), during a heating period from 7 January to 6 February 2022 using an air quality sensor Webber SP 86, at a fixed time, in the evening (6–8 PM). The measurements were taken at the height of 1.5 m above ground level. The detection limits for the sensor used were as follows: 0.1 μg/m^3^ for PM2.5 and PM10 and 0.001 mg/m^3^ for HCHO and TVOC. Typically, HCHO and TVOC are measured indoors. However, this time we decided to carry out the measurements outdoors.

## Health risk assessment

The health risk assessment associated with the presence of a harmful substance/element is usually based on estimating the magnitude of the risk and classifying it as carcinogenic or non-carcinogenic. Non-carcinogenic risk (HQ) is determined by comparing the calculated doses of pollutants which entered the body via individual exposure routes (in this case, inhalation) with the corresponding reference doses (RfD). In turn, carcinogenic risk (CR) is estimated as the lifetime probability of developing cancer as a result of exposure to a given carcinogen through a given route of exposure.

### Risk assessment for formaldehyde (HCHO)

Carcinogenic and non-carcinogenic risk assessment for HCHO was conducted using the methodology recommended by the US EPA^38–42^ To determine the value of health risk associated with inhalation exposure, inhalation exposure concentration (EC_inh_) was calculated using Eq. (1):

1 $${\text{EC}}_{{{\text{inh}}}} { } = {\text{C }} \cdot \frac{{{\text{ET}} \cdot {\text{EF}} \cdot {\text{ED}}}}{{{\text{AT}}}}\;[\upmu {\text{g}}/{\text{m}}^{{3}} ]$$

where EC_inh_—inhalation exposure concentration [μg/m^3^], C—concentration of HCOH in the air [μg/m^3^], ET—exposure time [hours/day], for adults 5 h/day and for children 2 h/day, EF—exposure frequency [days/year], 5 h and 2 h for 245 days (from October to May), ED—exposure duration [years], 70 and 6 years for adults and children, respectively, AT—averaging time [ED in years × 245 days/year × 5 h or 2 h/day in hours for non-carcinogens, 70 years × 245 days/year × 5 h/day in hours for carcinogens].

Carcinogenic risk (CR) associated with inhalation exposure was calculated using Eq. (2):

2 $${\text{CR }} = {\text{EC}}_{{{\text{inh}}}} \cdot {\text{IUR}}\left[ - \right]$$

where CR—carcinogenic risk [−], EC—exposure concentration [μg/m^3^], IUR—inhalational unit risk factor equal to 1.30 × 10^–5^ [μg/m^3^]^−1^^43^.

It is assumed that if CR < 10^–6^, the risk is low or negligible; if CR falls in the range of 10^–6^ and 10^–4^, the risk is uncertain, and if CR > 10^–4^, corrective measures must be taken^39,44,45^).

Non-carcinogenic inhalation risk for HCHO exposure was calculated as the Hazard Quotient (HQ) using Eq. (3).

3 $${\text{HQ }} = \frac{{{\text{EC}}_{{{\text{inh}}}} }}{{{\text{RfC}}_{{{\text{inh}}}} }} \left[ - \right]$$

where: HQ—hazard quotient [−], EC_inh_—exposure concentration [μg/m^3^], RfC_inh_—inhalational reference concentration, equivalent to: 9.83 μg/m^3^ for HCHO^46^.

It is accepted that at HQ ≤ 1 adverse health effects are unlikely, at HQ > 1 negative health effects are likely to occur.

## Results and discussion

### Air pollution (PM2.5, PM10, HCHO, TVOC) and the type of fuel combusted

Air quality measurements were carried out in the coldest months of the year, namely January and February (winter time). In the study area, this is the peak of the heating season, which determines air quality. The concentrations of PM2.5 and PM10 at the six sampling points ranged from 62 to 345 µg/m^3^ and from 75 to 369 µg/m^3^, respectively (Table 2). The levels of PM10 were higher than those of PM2.5 at nearly all sampling points. The finer fraction, i.e., PM2.5, accounted for an average of 91% of the PM10 fraction, which is consistent with the data published by Faria et al.^4^ and Yu et al.^47^. The coarse fraction also comprised larger particles, with a diameter of 2.5–10 µm, which are mineral particles lifted by wind from the ground. The proportion of these forms in the particulate matter in the winter time is small and, as shown in the present study, reaches only 9%. This result indicated, as expected, the dominant role of a point source of pollution, namely a chimney conveying exhaust gases and solid pollutants. The analysis showed that the impact of other sources of pollution in this area was small.

**Table 2:** Measured air quality parameters.

No	Symbols	Primary material	PM2.5	PM10	HCHO	TVOC
			[µg/m^3^]		[mg/m^3^]	
1		Wood of wet willow	122–152	138–199	0.010–0.230	0.001–0.005
2		Wood of dry willow	110–142	115–152	u.d.l.–0.020	0.010–0.016
3	AFw	Wood of acacia	112–133	116–148	u.d.l.–0.015	u.d.l.–0.006
4		Wood of oak	110–133	122–139	u.d.l.–0.002	u.d.l.–0.001
5		Wood of nut	126–153	129–156	0.011–0.063	u.d.l.–0.005
6		Coal I	130–152	151–168	u.d.l.–0.020	u.d.l.–0.002
7	CF	Coal II	126–155	130–155	0.001–0.004	u.d.l.–0.003
8		Coal III	104–118	128–141	0.011–0.012	u.d.l.–0.006
9		Coal pellets (ekogroszek) I	100–114	107–112	u.d.l.–0.004	u.d.l.–0.003
10		Coal pellets (ekogroszek) II	87–101	109–115	0.001–0.008	u.d.l.–0.001
11		Coal pellets (ekogroszek) III	93–119	117–133	u.d.l.–0.002	u.d.l.–0.001
12		Straw I	100–135	107–138	u.d.l.–0.010	u.d.l.–0.020
13	AF	Straw II	101–124	113–122	u.d.l.–0.004	u.d.l.–0.020
14		Wood pellets I	88–107	102–113	u.d.l.–0.005	u.d.l.–0.001
15		Wood pellets II	105–128	105–128	0.001–0.002	u.d.l.–0.004
16		Leaves of tree	93–111	103–119	0.001–0.010	u.d.l.–0.003
17		Green waste	106–132	120–144	0.001–0.004	u.d.l.–0.006
18		Mixed municipal wastes I	62–101	75–106	0.001–0.014	u.d.l.–0.001
19		Mixed municipal wastes II	111–199	80–106	0.005–0.015	u.d.l.–0.020
20	MMW	Mixed municipal wastes III	100–191	101–193	0.001–0.020	0.001–0.080
21		Mixed municipal wastes IV	91–150	88–135	0.004–0.016	u.d.l.–0.034
22		PCV packaginig (mineral oil)	137–251	150–257	0.002–0.033	u.d.l.–0.018
23		PCV packaging (plant prot. prod.)	126–237	151–256	0.002–0.062	0.010–0.095
24		PCV packaging (mix oil)	119–204	131–214	0.008–0.034	0.012–0.047
25		Plastic-coated paper cartons	80–111	120–136	u.d.l.–0.010	0.001–0.003
26	MW	Polystyrene	147–345	177–369	0.050–0.400	0.109–0.300
27		Rubber	125–301	155–338	0.002–0.159	0.030–0.201
28		Imitation leather	102–238	210–243	u.d.l.–0.016	0.001–0.004
29		Waste paper	76–101	95–135	u.d.l.–0.005	0.001–0.002
30		Sponges	145–265	200–303	0.001–0.309	0.030–0.070
31		Textiles	129–278	147–294	0.001–0.018	u.d.l.–0.002
32		Plywood	103–178	148–201	u.d.l.–0.003	u.d.l.–0.072
For all material and measured points (*n* = *32* × *6*)		Min	62	75	u.d.l	u.d.l
		Max	345	369	0.400	0.300
		Me	122	135	0.004	0.002
		Av. ± SD	136 ± 50	152 ± 57	0.021 ± 0.051	0.017 ± 0.045

**Fig. 2:**
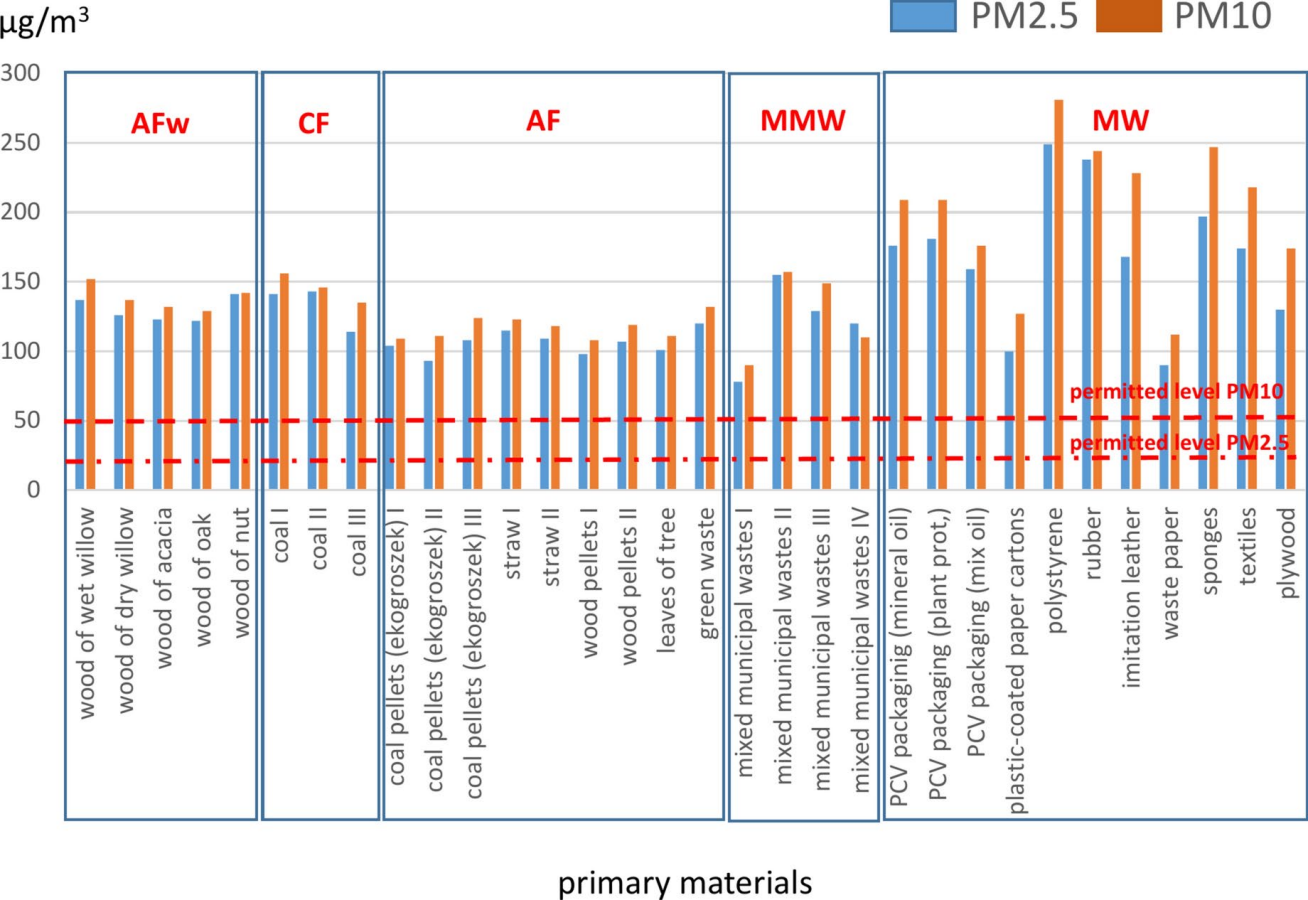
Average contents of PM2.5 and PM10 in the air during the combustion of conventional (CF) and alternative fuels (AFw, AF) and municipal waste (MW, MMW).

The obtained values (Table 2) significantly exceeded the limit values for PM2.5 and PM10 in the air set out for the protection of human health^20,48,49^, for the 24-h average, i.e., 20 and 50 µg/m^3^, for all materials (fuels) at all sampling points. In the case of PM2.5, the levels were exceeded 3.1–17.2 times and in the case of PM10, 0.5–7.4 times (Fig. 2). The situation is very concerning, especially with respect to PM2.5. Particulates with a diameter smaller than 2.5 µm are atmospheric aerosols considered most harmful to human health, as they can easily and directly penetrate into the trachea or bronchi and consequently enter the bloodstream. Respirable dust particles play a role in the exacerbation of asthma, impaired lung function, lung, throat and larynx cancers, cardiac arrhythmias, vasculitis, atherosclerosis and low birth weight and respiratory problems in babies exposed to these particulates during fetal development^4,6^. They have also been reported to exacerbate circulatory and respiratory symptoms in people suffering a long-term exposure to these particulates^10^. In turn, PM10 are a mixture of suspended particles, whose diameter does not exceed 10 µm. Their detrimental effect is associated with the presence of other harmful substances, i.e. benzopyrenes, furans or dioxins, also in the form of potentially toxic elements (Zn, Tl, As, Pb, Cd or Hg). They negatively affect the respiratory system, being responsible for coughing attacks, wheezing, rapid bronchitis or the development of asthma. A study by Park et al.^7^ demonstrated that a long-term exposure to PM10 indirectly increases the risk of heart attack and stroke.

As already mentioned, the obtained values indicate that PM2.5 and PM10 levels exceeded the limit values at all sampling points and for all materials, regardless of whether these were conventional or alternative fuels (Fig. 2). The average PM2.5 and PM10 levels in the air after the combustion of individual groups of fuels decreased in the following order (PM2.5, PM10 – data in µg/m^3^): selected fractions of municipal waste (169, 202) > conventional fuels – hard coal (133, 146) > wood – various species (130, 138) > mixed stream of municipal waste (121, 127) > alternative fuels – various types (106, 117).

The second group of air quality parameters measured were organic pollutants, i.e., formaldehyde (HCHO) and volatile organic compounds (TVOC). The first one—formaldehyde (methanal), is an organic compound formed during the incomplete combustion of carbon-containing substances. It is the key compound among the oxidized forms of volatile organic substances found indoors due to its presence in numerous materials (including varnishes, paints, and adhesives) and even preservatives. Therefore, multiple materials are saturated with formaldehyde. Our measurements showed that at all sampling points the concentration of this compound did not exceed 0.400 mg/m^3^, which is the odor detection threshold. In concentrations exceeding 0.01 ppm, formaldehyde may cause irritation of the epidermis, nasopharyngeal mucosa and eyes. According to the guidelines by the German Ministry of the Environment^50^, formaldehyde levels should not exceed 0.1 mg/m^3^ in indoor air.

Volatile organic compounds (TVOC), the other indicator measured, have a high tendency to evaporate (especially at low temperatures), causing gaseous air pollution^51,52^. Our study showed that the air levels of TVOC levels ranged from trace amounts (< 0.001 mg/m^3^) to max. 0.3 mg/m^3^ (Table 2). It is believed that concentrations below 0.2 mg/m^3^ do not cause irritation or poor well-being, whereas levels in the range of 0.2–3.0 mg/m^3^ are conducive to irritation and well-being deterioration^50^. The WHO established an indoor air quality guideline of 0.1 mg/m^3^for a 30-min exposure^49^.

**Fig. 3:**
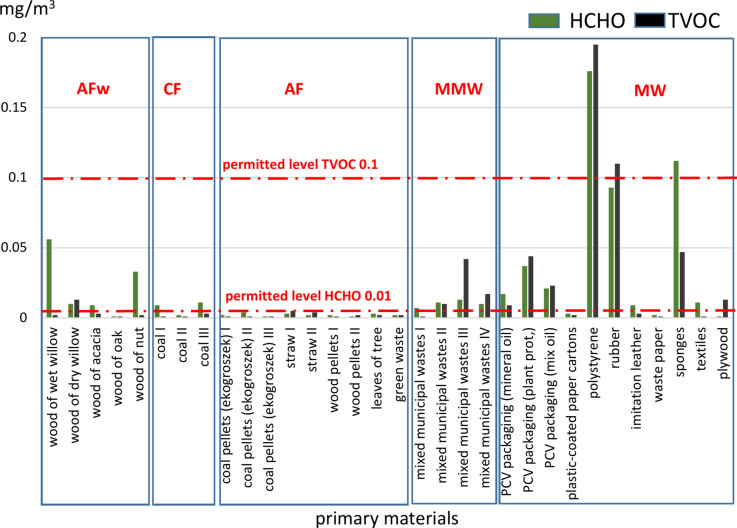
Average contents of HCHO and TVOC in the air during the combustion of conventional (CF) and alternative fuels (AFw, AF) and municipal waste (MW, MMW).

Thus, HCHO and TVOC concentrations at selected sampling points exceeded the limit values (i.e., 0.01 and 0.02 mg/m^3^, respectively). In the case of HCHO, this pertained to wood (different species), mixed municipal waste and some groups of municipal waste (i.e., PVC packaging, polystyrene, rubber and upholstery foam). As for TVOC, the limit values were exceeded in the case of mixed municipal waste and some fractions of municipal waste, i.e., PVC packaging, polystyrene, rubber, upholstery foam and furniture boards (Fig. 3). Some countries have set out standards for the limit values of TVOC, but these apply to indoor air quality. For example, the Chinese limit value for TVOC is 0.6 mg/m^3^^53^, while relevant legislation in Germany specifies the following HCOH and TVOC concentrations (ppm): 0.001–0.4 and 0.3–1.2, respectively.

The average HCHO and TVOC levels in the air after the combustion of individual groups of fuels decreased in the following order (HCHO, TVOC – data in mg/m^3^): separate fractions of municipal waste (0.044, 0.041) > mixed stream of municipal waste (0.022, 0.018) > wood – different types (0.010, 0.005) > conventional fuels – hard coal (0.007, 0.002) > alternative fuels – different types (0.002, 0.002).

Having analyzed the impact of the combustion of conventional and alternative fuels on the amount of inorganic pollutants in the air, we found that CF generated the highest concentrations of PM2.5 and PM10 (133 and 146 µg/m^3^, respectively). In turn, the combustion of AF, such as coal pellets, straw, green waste or wood pellets, generated PM2.5 and PM10 amounts that were approximately 20% lower compared to CF (106 and 117 µg/m^3^, respectively). A slightly different situation was observed for wood combustion (various types). Here, the PM2.5 and PM10 values were only slightly lower, by about 2% (130 and 138 µg/m^3^, respectively), than the results recorded for CF. For the wet wood sample, the values of PM2.5 and PM10 in the air were 10% higher than for the wood sample of the same species, but dried out.

As for organic pollutants, the highest concentrations in the air, both for HCHO and TVOC, were recorded after burning various wood species (AFw). The average concentrations of these parameters recorded for this entire group of fuels were 0.022 and 0.004 mg/m^3^, respectively, being 10- and twofold higher than the concentrations observed for other AF, and 3- and twofold higher than those recorded for CF. These relationships stem from the chemical composition of the primary materials. This is because the main mass of wood is comprised of organic substances consisting of 4 main elements: C (about 49.5%), H (6.3%) and O_2_ and N (44.2% in total, with the share of N being about 0.12%). The proportion of mineral matter is low and depends on the species of wood, but does not exceed 0.2–1.7 wt%^54^. In contrast, hard coal contains from 75% to as much as 97% of C, while other elements such as: H (2–6%), O2 (1–18%), N (0.5–2%) and S (0.2–2%) make up the rest of its chemical composition^55,56^. The high levels of particulate matter (PM2.5 and PM10) may be due to the high content (up to 40%) of mineral matter (ash – the residue from incomplete combustion), which is transferred to the external environment through the chimney^57^. The chemical composition of ash (composed mainly of Al, Fe, Si, Ca, Na, K, and S oxides and elements described as potentially toxic: Cu, Pb, Cd, Hg) and its effect on human health as well as environmental pollution have been the subject of numerous scientific studies^58–62^.

These differences have direct health implications for individuals breathing polluted air, especially in the long term. In terms of pollutant emissions (organic and inorganic) and their impact on human health, the least harmful fuels burned in household furnaces are coal pellets and wood pellets.

In summary, the combustion of individual fuel groups and the obtained PM2.5, PM10, HCOH and TVOC values showed that:

- the highest average concentrations (for all materials analyzed *n* = 32) were observed during the combustion of polystyrene; these were 249 and 281 µg/m^3^ (PM2.5, PM10) and 0.176 and 0.195 mg/m^3^ (HCOH, TVOC),
- in the MMW group, the highest concentrations were recorded for sample no. III (155, 149, 0.013 and 0.042, respectively); this was mixed waste collected in a detached house inhabited by two adults who frequently shop online,
- in the AF group, the highest concentrations were found during the combustion of green waste (120, 132, 0.004, 0.004, respectively); its main constituents were dry kitchen waste and shrub and grass cuttings,
- in the AFw group, the highest concentrations of PM2.5, PM10 and HCOH were recorded during the combustion of wet willow wood (152, 166 µg/m^3^ and 0.23 mg/m^3^, respectively). For the sample of the same wood, but dried out, these values were on average 20% lower,
- in the CF group, for the two hard coal samples analyzed, the highest concentrations of PM2.5 and PM10 were 155 and 168 µg/m^3^, respectively, and those for HCOH and TVOC were 0.011 and 0.003 mg/m^3^, respectively.

### Airborne dispersion of pollutants

A chimney discharging exhaust gases from a furnace (boiler), where the fuel combustion process takes place, is a typical point source of pollution. The airborne dispersion of harmful substances contained in the plume depends on the height reached by the exhaust gases, the size of their particles and climatic factors, i.e., atmospheric stability, humidity, and wind direction and speed^63,64^. The substances are mainly transported by wind and mix with the surrounding air, with the scattering taking place horizontally (this process occurs much faster and depends on the topography of the area) and vertically^65,66^.

To analyze air pollutant dispersion from a point source, we employed the Gaussian Dispersion Model, which uses a normal distribution curve (Gaussian distribution) to describe the concentration of harmful substances. The variables included in the analysis were the emission rate from the source and wind speed. Ground level concentration was calculated using Eq. (4).

4 $$C\left( {x,y} \right) = \frac{Q}{{\pi \mu \sigma_{y} \sigma_{z} }}\exp \left( {\begin{array}{*{20}c} { - H^{2} } \\ {2\sigma_{z}^{2} } \\ \end{array} } \right)\exp \left( {\begin{array}{*{20}c} { - y^{2} } \\ {2\sigma_{y}^{2} } \\ \end{array} } \right)$$

where C (*x,y*)—concentration at ground level at point *x,y* [µg/m^3^], x—wind-blown distance [m], y—horizontal distance from the source (symmetrical chimney plume) [m], Q—emission rate of the substance [µg/s], H—effective chimney height [m], µ—average wind speed [m/s], σ_y_—horizontal scattering coefficient (standard deviation)), σ_z_—vertical scattering coefficient (standard deviation).

In our study, we analyzed the concentrations of the substances in the air, but not at ground level, which is undoubtedly important in soil research, but at the level of a person’s mouth and nose (i.e., 1.5 m). The selection of the sampling points has been described in the previous subsection.

**Fig. 4:**
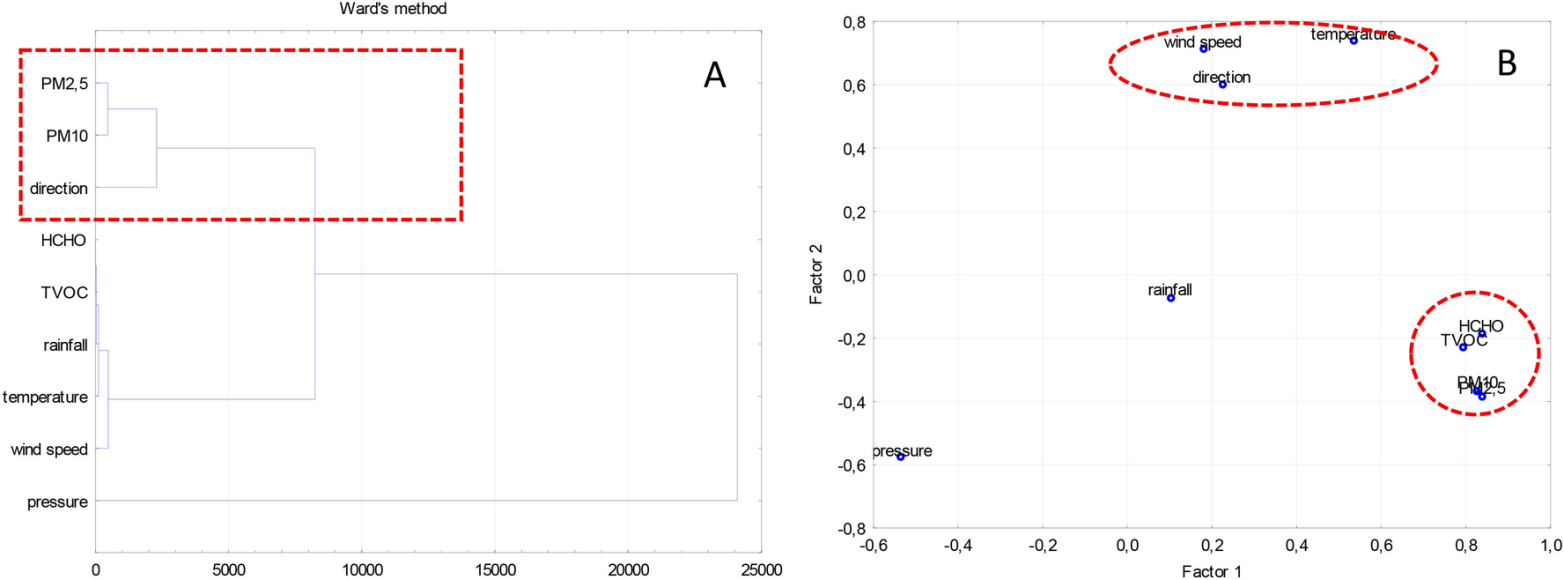
Dendrogram (**A**) and factor analysis (**B**) for the analyzed pollutants and climatic factors.

At the initial stage of the analysis, we verified whether there were any associations between the factors studied (Table 3, Fig. 4). The statistical analyses (i.e., correlation coefficients, cluster analysis and factor analysis) indicated that:

- atmospheric factors (such as wind speed and direction and temperature) play a major role in the dispersion of pollutants (Fig. 4B),
- wind direction plays a major role in PM2.5 and PM10 dispersion (Fig. 4A), which is consistent with the findings obtained by Galán-Madruga et al.^66^,
- there is a very strong correlation between the concentrations of PM2.5 and PM10 (*r* = 0.88),
- there is a very strong correlation between the concentrations of HCHO and TVOC (*r* = 0.68), and between organic pollutants (HCHO and TVOC) and particulate matter pollutants (Tab. 4), *r* = 0.67 and 0.64 for PM2.5 and *r* = 0.63 and 0.60 for PM10, respectively.

**Table 3:** Correlation coefficient between measured parameters.

*r*	Distance	PM2.5	PM10	HCHO	TVOC	Wind direction	Wind speed	Pressure	Temperature	Precipitation
Distance	1.00	− 0.20	− 0.13	− 0.19	− 0.12	0.00	0.00	0.00	0.00	0.00
PM2.5		1.00	***0.88***	*0.67*	*0.64*	0.04	− 0.13	− 0.18	0.18	0.00
PM10			1.00	*0.63*	*0.60*	0.03	− 0.16	− 0.19	0.23	− 0.02
HCHO				1.00	*0.68*	0.03	0.09	− 0.31	0.24	0.09
TVOC					1.00	0.05	0.08	− 0.23	0.17	0.15
Wind direction						1.00	0.23	− 0.18	0.57	− 0.32
Wind speed							1.00	− 0.42	0.46	0.04
Pressure								1.00	− *0.68*	− 0.20
Temperature									1.00	0.02
Precipitation										1.00

If:

*0.5* ≤ *r* < *0.7 high correlation.*

***0.7* ≤ *r* < *0.9**** very high correlation.*

**0.9 ≤ *r* < 1.0** almost certain correlation.

Significant values are in italic and bolditalic

**Table 4:**
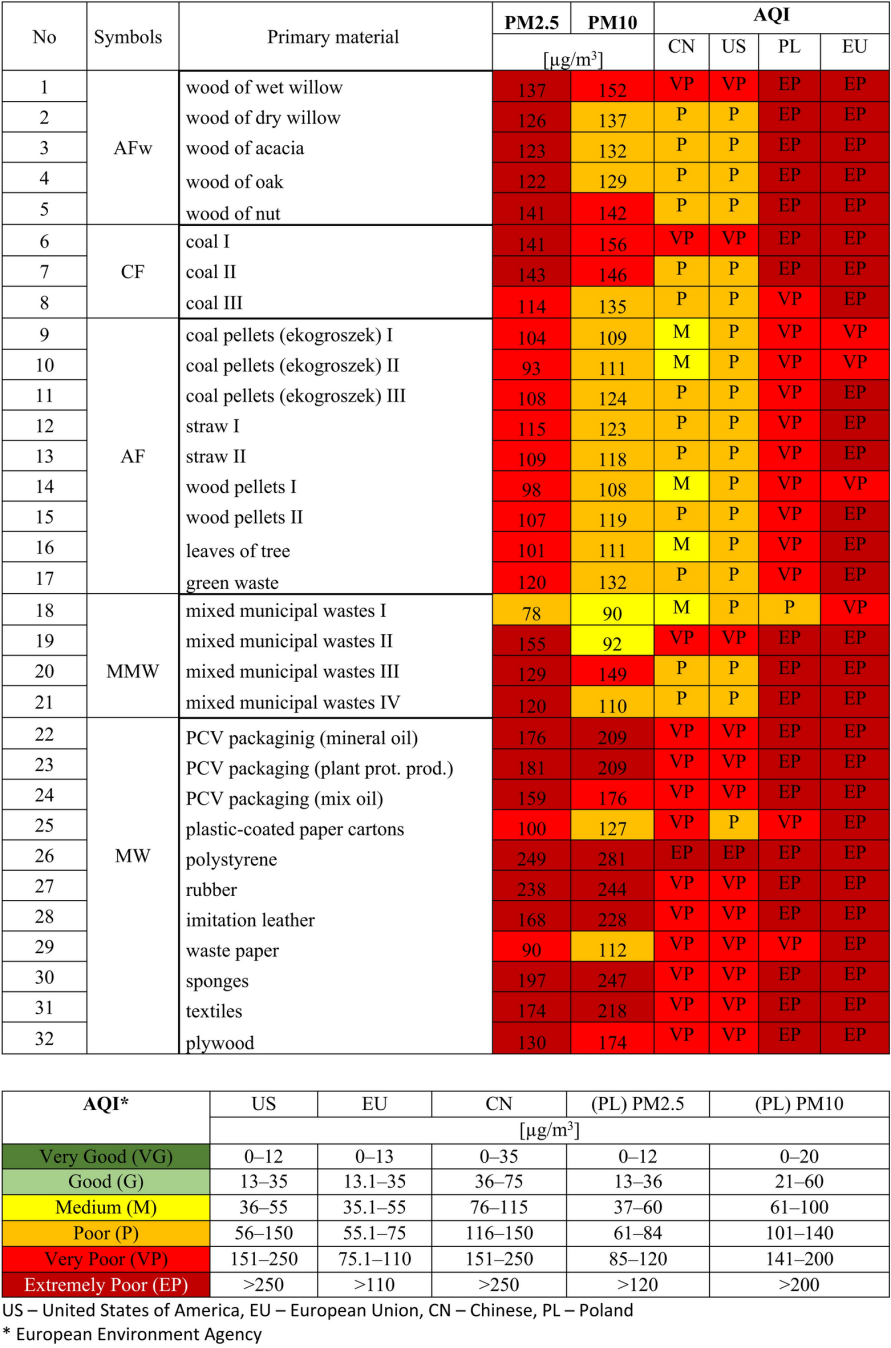
Air quality index calculated for analyses materials.

**Fig. 5:**
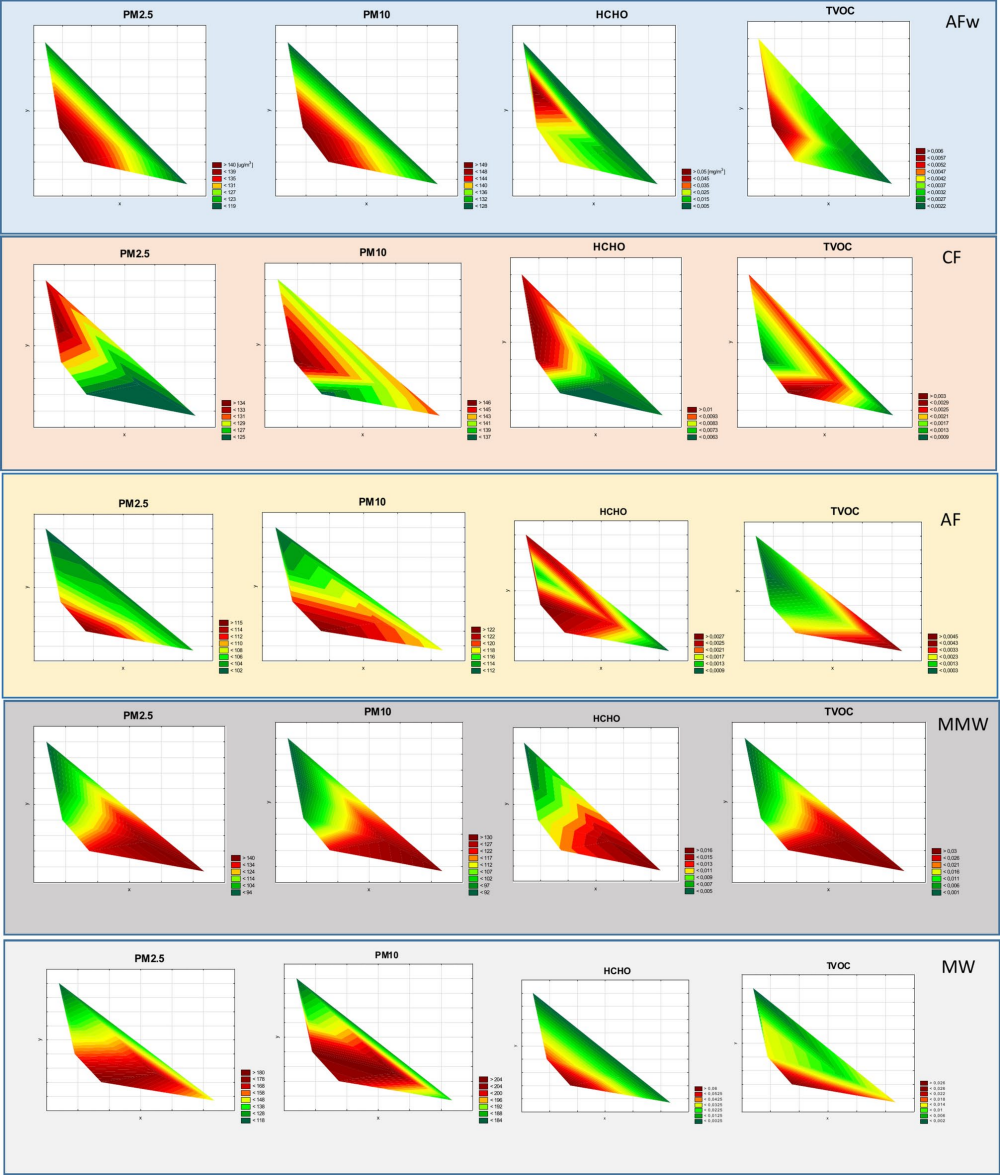
Spread of pollutants in the atmosphere (assumed average values calculated for all samples from a particular group: AFw *n* = *5*, CF *n* = *3*, AF *n* = *9*, MMW *n* = *4*, MW *n* = *11,* see Tab. 1).

We analyzed pollutant dispersion for all the substances combusted (Fig. 5S). The analysis revealed that:

- under the same atmospheric conditions, particulate pollutants (PM2.5 and PM10) disperse somewhat differently than organic pollutants (TVOC and HCHO). Organic pollutant plumes are considerably smaller and narrower (samples 1, 2, 7, 8, 12, 16, 25, 26, 31, and 32) as compared to particulate matter plumes,
- PM2.5 and PM10 dispersion plumes for the majority of the materials analyzed are very similar (Fig. 5S),
- lack of wind is conducive to the formation of fog, i.e., no clear plume is observed (samples 27, 30),
- wind speed > 30 km/h causes rapid pollutant dispersion and the formation of a plume consistent with the predominant wind direction (samples 1, 2),
- organic pollutant dispersion is largely affected by low (negative) temperatures, which considerably enlarge the TVOC and HCHO dispersion plumes (samples 19, 20, 21).

Distributions of average pollutant concentrations emitted as a result of combusting all groups of the conventional, alternative and municipal waste fuels analyzed in the study have been presented in Fig. 5. Based on this data, we found that in the case of:

- CF and AF, the plumes for all the substances analyzed differed substantially, which may stem from the large variation between individual coal samples (CF) and differences between pollutant emissions during the combustion of such materials as coal pellets, straw or green waste (AF),
- AFw, there was a difference between the organic pollutant plume and the particulate matter pollutant plume; in the case of the latter, the plume was much smaller (by ~ 20–50%) and narrower, with a slightly elongated shape (perpendicular to the predominant wind direction),
- MMW, the organic and particulate matter pollutant plumes were nearly identical and their direction was consistent with the predominant wind direction (SW); this is undoubtedly related to the stability of the atmosphere and the lowest average temperature (− 10 °C) recorded during the measurements,
- selected fractions of MW, the organic and particulate matter pollutant plumes were very similar.

### Health risk associated with inhalation of polluted air

Health risk assessment relies on several key assumptions. These are based on toxicological testing carried out for individual substances^43^. Due to the fact that we did not measure the concentrations of individual constituents of PM2.5, PM10 and TVOC, and the registered values pertain to the entire group of compounds, it was not possible to carry out a full health risk assessment using generally accepted equations. This analysis could only be performed for formaldehyde (HCHO). Nevertheless, to assess the impact of the other parameters on the health of local inhabitants, we used the Air Quality Index (AQI) and the recommended limit values.

#### Formaldehyde

We calculated the carcinogenic health risk associated with the presence of formaldehyde in the air, following the combustion of various fuels, for two age groups: children and adults (“Health Risk Assessment” section). As mentioned in the method description, we assumed that children spend only 2 h a day outside during the heating season, whereas adults 5 h a day, which is related to e.g., the need to remove snow from the area around homes and to perform agricultural work associated with the late (autumn) harvesting of agricultural crops.

The obtained CR values for children and adults at all sampling points ranged as follows: 7.80E-04–1.04E-01 and 6.50E-05–2.60E-02, respectively (Table S1). Values denoting low or negligible risk (CR < 10^–6^) were not observed for any of the substances combusted (although at several points, the level of pollutants was below the detection limit of the sensor). In turn, CR of 10^–6^–10^–4^ (uncertain risk) accounted for 77% of the values obtained for adults and 47.5% for children. Substances posing an uncertain risk during combustion were oak wood, straw, coal and wood pellets and green waste. CR > 10^–4^ denoting an unacceptable carcinogenic risk accounted for 23% and 52% of the values obtained for adults and children, respectively. These values pertained to the combustion of mixed municipal waste (samples 19 and 21) and PVC packaging, polystyrene, rubber, artificial leather and textiles. The combustion of polystyrene and rubber posed the highest health risk for adults. We found that the combustion of polystyrene generated the highest CR values of 1.04E-01 (children) and 2.60E-02 (adults). Such high values not only call for corrective action, but also point to the need of expanding research about the impact of this practice on other environmental components^67^.

Non-carcinogenic risk (HQ) calculated for formaldehyde fell within the range of 0.51–203 (adults) and 0.51–814 (children). Only 33% and 15% of the values (for adults and children, respectively) met the methodologically assumed recommendation of HQ ≤ 1. It is concerning that 2% and 11% of the HQ values exceeded 100 and, in the case of children, 1% of the values exceeded 500. This occurred during the combustion of polystyrene and upholstery foam. This is not an unprecedented result in the scientific literature. Values as high as these (HQ = 214.2 for < 2 year-old children and HQ = 51.8 for 2–6 year-old children) have been reported by Li et al.^68^, who studied HCOH in indoor environments.

#### TVOC

We compared our measurements of TVOC concentrations in the air with relevant legal recommendations. However, the proposed limit values for TVOC refer to indoor air quality^69^. The first limit value 0.3 mg/m^3^ was proposed by Seifert^70^. However, it had not been determined based on toxicological studies. Another limit value was proposed in 2007^69^. It was extended in terms of assessing the level of pollution with these compounds, as it presented five pollution levels and specific recommendations to be taken when such pollution occurs. However, it still did not address health risks.

Referring to these 5 levels of pollution—and their corresponding limit values of (mg/m^3^): < 0.3 (level I), 0.3–1(level II), > 1–3 (level III), > 3–10 (level IV) and > 10–25 (level V), we determined that the maximum value of 0.3 mg/m^3^ points to a minimal presence of volatile organic compounds and the situation does not require any corrective measures to be taken. The Chinese legislation^53^ recommends the value of 0.6 mg/m^3^, which is considerably (two times) higher.

#### Air quality index—PM2.5 and PM10

The next step in the study involved the parametric assessment of air quality at the six sampling points. For this purpose, we calculated the Air Quality Index (AQI), as it is the easiest way to determine air pollution levels. The AQI values range between 0 and 500, and the higher the index, the more polluted the air. Our assessment was based on the particulate matter levels (PM2.5 and PM10), but it can also include other pollutants, i.e., SOx, NOx, CO, COx and O_3_, C_6_H_6_. AQIs for individual pollutants are calculated based on hourly concentrations of these compounds and assigned to a relevant category presented in the table of AQI ranges (Table 4). Next, the overall index takes the value of the worst individual index among the pollutants measured.

The analysis showed that according to the Polish and European classification, air quality (AQI) during the combustion of all the materials analyzed was very poor (*n* = *12*) or extremely poor (*n* = *19*) (Tab. 4). The exception was a sample of materials marked with number 18 (mixed municipal waste I), whose combustion resulted in medium AQI. Very poor AQI was obtained for all alternative fuels (coal pellets, straw, wood pellets, leaves and green waste). This means that pollution generated by burning these materials is so high that patients, the elderly, pregnant women, and small children should avoid staying outside and other people should minimize outdoor activity. Long-term inhalation exposure to air of quality like this increases the risk of changes in the respiratory, cardiovascular and immune systems.

Extremely poor AQI was found for all species of wood and the majority of coals and mixed municipal waste. Extremely poor air quality was also observed for nearly all fractions of municipal waste, except for paper and cardboard packaging, whose combustion resulted in very poor air quality. Inhalation exposure to very poor air quality can lead to health problems, such as disorders of the respiratory, cardiovascular and immune systems. Outdoor activities should be limited to the necessary minimum.

The American (US values, Table 4) and Chinese legislation (CN values) is slightly different from the legal solutions adopted in Europe, and thus in Poland (which is an EU Member State), in terms of measurement ranges. Of these, the EU regulations are the most restrictive. The good, fair and moderate AQI ranges are identical in the US and EU law, but significantly different in the CN legislation. In turn, the EU legislation is much more restrictive with regard to the poor, very poor and extremely poor AQI ranges as compared to the provisions in the US and CN (which are nearly identical), which is reflected in the final AQI assessment.

Air quality determined based on the CN criteria would be medium for 5 samples, poor for 13 samples, very poor for 13 samples and extremely poor for 1 sample (Table 4). According to the US criteria, AQI would be poor for 19 samples, very poor for 12 samples and extremely poor for 1 sample. As for the EU legislation (assigning the class according to the highest value of any parameter), AQI would be very poor for 4 samples and extremely poor for the other 28 samples.

The general conclusion stemming from this data is that all the solid fuels (hard coal), alternative fuels (coal pellets, pellets, wood) and municipal waste (mixed or selected fractions) analyzed in the study emit substantial amounts of PM2.5 and PM10 when combusted in household furnaces, which results in very poor or extremely poor air quality in the surrounding area. It should be stressed that the study had several limitations such as the existing climatic conditions, the terrain and the selected groups of materials (n = 32) subjected to combustion. Undoubtedly, the parameters of the furnace used in the study also significantly determined the study results.

## Conclusion

In light of the research hypotheses, we found that:

- each type of fuel (AF, CF, AFw) combusted in a household furnace is a source of air pollutants, especially fine PM2.5 particles, which are harmful to human health,
- combustion of municipal waste in household furnaces is a substantial source of organic air pollutants (TVOC and HCHO) and thus constitutes a dangerous exposure source to people living in nearby areas,
- it is vital to stop burning such materials as polystyrene, rubber and upholstery foam in household furnaces due to the drastically high health risk associated with this practice,
- in rural areas, solid fuel combustion is a significant factor deteriorating the chemical condition of soils, especially those used for agricultural purposes.
- Furthermore, the study showed that research on this subject should be continued and extended to include detailed identification of individual particulate matter and VOC constituents, which would allow for calculating the overall health risk.

Despite the existing knowledge and legal regulations, the economic and political situation may prompt people to use waste for energy purposes. The data presented in the paper undoubtedly make an important contribution to the discussion on expanding and increasing the share of renewable sources in energy production in rural areas. The solution to the existing situation is to develop a model for managing renewable energy resources in such a way that individual energy sources are used most efficiently, with the least social cost and in accordance with the assumptions of sustainable development and environmental policies.

## Supplementary Information

Supplementary Information 1.

Supplementary Information 2.

## Data Availability

The datasets used and/or analysed during the current study available from the corresponding author on reasonable request.
