# Supplementary material for: Burning of municipal waste in household furnaces and the health of their owners
Source: Sci Rep. 2024 Dec 30;14:32011. doi: 10.1038/s41598-024-83572-4 (PMC11686134; doi:10.1038/s41598-024-83572-4)
Supplement: Supplementary file 1 — Supplementary Information 1. [file 41598_2024_83572_MOESM1_ESM.docx]

SW ↘ (37.25 km/h)

W → (30 km/h)

W → (22.75 km/h)

NW ↗ (14.25 km/h)

W → (22.75 km/h)

W → (21.5 km/h)

W → (21.5 km/h)

W → (24.5 km/h)

W → (24.5 km/h)

W → (14.25 km/h)

W → (38.25 km/h)

W → (12.75 km/h)

W → (12.75 km/h)

W → (27.25 km/h)

W → (12.25 km/h)

NW → (22.00 km/h)

W → (46.00 km/h)

NE → (18.00 km/h)

S → (9.25 km/h)

S → (9.25 km/h)

S → (9.25 km/h)

SW → (0.00 km/h)

SW → (0.00 km/h)

SW → (0.00 km/h)

W → (7.00 km/h)

W → (46.00 km/h)

W → (0.00 km/h)

SW → (9.00 km/h)

SE → (13.00 km/h)

SW → (0.00 km/h)

SE → (13.00 km/h)

SW → (0.00 km/h)

Fig. 5S. Waffle charts made for each type of pollutant analyzed, at measurement points 1-6.
