# Supplementary material for: Burning of municipal waste in household furnaces and the health of their owners
Source: Sci Rep. 2024 Dec 30;14:32011. doi: 10.1038/s41598-024-83572-4 (PMC11686134; doi:10.1038/s41598-024-83572-4)
Supplement: Supplementary file 2 — Supplementary Information 2. [file 41598_2024_83572_MOESM2_ESM.docx]

| Table S1. Risk assessment calculation for formaldehyde | | | | | | | | |
| --- | --- | --- | --- | --- | --- | --- | --- | --- |
| No. | **Point**  **(fig. 1)** | **HCHO**  **µg/m^3^** | **ECinh (CR/Ad)** | **ECinh (CR/Ch)** | **CR**  **Adults** | **CR**  **Children** | **HQ**  **Adults** | **HQ**  **Children** |
| 1 | **1** | 30.000 | 150.00 | 600.00 | 1.95E-03 | 7.80E-03 | 15.26 | 61.04 |
|  | **2** | 21.000 | 105.00 | 420.00 | 1.37E-03 | 5.46E-03 | 10.68 | 42.73 |
|  | **3** | 10.000 | 50.00 | 200.00 | 6.50E-04 | 2.60E-03 | 5.09 | 20.35 |
|  | **4** | 33.000 | 165.00 | 660.00 | 2.15E-03 | 8.58E-03 | 16.79 | 67.14 |
|  | **5** | 230.000 | 1150.00 | 4600.00 | 1.50E-02 | 5.98E-02 | 116.99 | 467.96 |
|  | **6** | 10.000 | 50.00 | 200.00 | 6.50E-04 | 2.60E-03 | 5.09 | 20.35 |
|  | **av.** | 56 | 278.33 | 1113.33 | 3.62E-03 | 1.45E-02 | 28.31 | 113.26 |
| 2 | **1** | 20.000 | 100.00 | 400.00 | 1.30E-03 | 5.20E-03 | 10.17 | 40.69 |
|  | **2** | 14.000 | 70.00 | 280.00 | 9.10E-04 | 3.64E-03 | 7.12 | 28.48 |
|  | **3** | 7.000 | 35.00 | 140.00 | 4.55E-04 | 1.82E-03 | 3.56 | 14.24 |
|  | **4** | 17.000 | 85.00 | 340.00 | 1.11E-03 | 4.42E-03 | 8.65 | 34.59 |
|  | **5** | u.d.l. | u.d.l. | u.d.l. | - | - | - | - |
|  | **6** | u.d.l. | u.d.l. | u.d.l. | - | - | - | - |
|  | **av.** | 10 | 48.33 | 193.33 | 6.28E-04 | 2.51E-03 | 4.92 | 19.67 |
| 3 | **1** | 15.000 | 75.00 | 300.00 | 9.75E-04 | 3.90E-03 | 7.63 | 30.52 |
|  | **2** | 8.000 | 40.00 | 160.00 | 5.20E-04 | 2.08E-03 | 4.07 | 16.28 |
|  | **3** | 8.000 | 40.00 | 160.00 | 5.20E-04 | 2.08E-03 | 4.07 | 16.28 |
|  | **4** | 20.000 | 100.00 | 400.00 | 1.30E-03 | 5.20E-03 | 10.17 | 40.69 |
|  | **5** | 4.000 | 20.00 | 80.00 | 2.60E-04 | 1.04E-03 | 2.03 | 8.14 |
|  | **6** | u.d.l. | u.d.l. | u.d.l. | - | - | - | - |
|  | **av.** | 9 | 45.83 | 183.33 | 5.96E-04 | 2.38E-03 | 4.66 | 18.65 |
| 4 | **1** | 2.000 | 10.00 | 40.00 | 1.30E-04 | 5.20E-04 | 1.02 | 4.07 |
|  | **2** | 1.000 | 5.00 | 20.00 | 6.50E-05 | 2.60E-04 | 0.51 | 2.03 |
|  | **3** | u.d.l. | u.d.l. | u.d.l. | - | - | - | - |
|  | **4** | 2.000 | 10.00 | 40.00 | 1.30E-04 | 5.20E-04 | 1.02 | 4.07 |
|  | **5** | u.d.l. | u.d.l. | u.d.l. | - | - | - | - |
|  | **6** | u.d.l. | u.d.l. | u.d.l. | - | - | - | - |
|  | **av.** | 1 | 4.17 | 16.67 | 5.42E-05 | 2.17E-04 | 0.42 | 1.70 |
| 5 | **1** | 80.000 | 400.00 | 1600.00 | 5.20E-03 | 2.08E-02 | 40.69 | 162.77 |
|  | **2** | 12.000 | 60.00 | 240.00 | 7.80E-04 | 3.12E-03 | 6.10 | 24.42 |
|  | **3** | 11.000 | 55.00 | 220.00 | 7.15E-04 | 2.86E-03 | 5.60 | 22.38 |
|  | **4** | 63.000 | 315.00 | 1260.00 | 4.10E-03 | 1.64E-02 | 32.04 | 128.18 |
|  | **5** | 23.000 | 115.00 | 460.00 | 1.50E-03 | 5.98E-03 | 11.70 | 46.80 |
|  | **6** | 11.000 | 55.00 | 220.00 | 7.15E-04 | 2.86E-03 | 5.60 | 22.38 |
|  | **av.** | 33 | 166.67 | 666.67 | 2.17E-03 | 8.67E-03 | 16.95 | 67.82 |
| 6 | **1** | 1.000 | 5.00 | 20.00 | 6.50E-05 | 2.60E-04 | 0.51 | 2.03 |
|  | **2** | u.d.l. | u.d.l. | u.d.l. | - | - | - | - |
|  | **3** | u.d.l. | u.d.l. | u.d.l. | - | - | - | - |
|  | **4** | 20.000 | 100.00 | 400.00 | 1.30E-03 | 5.20E-03 | 10.17 | 40.69 |
|  | **5** | 20.000 | 100.00 | 400.00 | 1.30E-03 | 5.20E-03 | 10.17 | 40.69 |
|  | **6** | 15.000 | 75.00 | 300.00 | 9.75E-04 | 3.90E-03 | 7.63 | 30.52 |
|  | **av.** | 9 | 46.67 | 186.67 | 6.07E-04 | 2.43E-03 | 4.75 | 18.99 |
| 7 | **1** | 3.000 | 15.00 | 60.00 | 1.95E-04 | 7.80E-04 | 1.53 | 6.10 |
|  | **2** | 3.000 | 15.00 | 60.00 | 1.95E-04 | 7.80E-04 | 1.53 | 6.10 |
|  | **3** | 2.000 | 10.00 | 40.00 | 1.30E-04 | 5.20E-04 | 1.02 | 4.07 |
|  | **4** | 4.000 | 20.00 | 80.00 | 2.60E-04 | 1.04E-03 | 2.03 | 8.14 |
|  | **5** | 1.000 | 5.00 | 20.00 | 6.50E-05 | 2.60E-04 | 0.51 | 2.03 |
|  | **6** | 1.000 | 5.00 | 20.00 | 6.50E-05 | 2.60E-04 | 0.51 | 2.03 |
|  | **av.** | 2 | 11.67 | 46.67 | 1.52E-04 | 6.07E-04 | 1.19 | 4.75 |
| 8 | **1** | 11.000 | 55.00 | 220.00 | 7.15E-04 | 2.86E-03 | 5.60 | 22.38 |
|  | **2** | 12.000 | 60.00 | 240.00 | 7.80E-04 | 3.12E-03 | 6.10 | 24.42 |
|  | **3** | 11.000 | 55.00 | 220.00 | 7.15E-04 | 2.86E-03 | 5.60 | 22.38 |
|  | **4** | 11.000 | 55.00 | 220.00 | 7.15E-04 | 2.86E-03 | 5.60 | 22.38 |
|  | **5** | 12.000 | 60.00 | 240.00 | 7.80E-04 | 3.12E-03 | 6.10 | 24.42 |
|  | **6** | 11.000 | 55.00 | 220.00 | 7.15E-04 | 2.86E-03 | 5.60 | 22.38 |
|  | **av.** | 11 | 56.67 | 226.67 | 7.37E-04 | 2.95E-03 | 5.76 | 23.06 |
| 9 | **1** | 4.000 | 20.00 | 80.00 | 2.60E-04 | 1.04E-03 | 2.03 | 8.14 |
|  | **2** | 4.000 | 20.00 | 80.00 | 2.60E-04 | 1.04E-03 | 2.03 | 8.14 |
|  | **3** | 4.000 | 20.00 | 80.00 | 2.60E-04 | 1.04E-03 | 2.03 | 8.14 |
|  | **4** | u.d.l. | u.d.l. | u.d.l. | - | - | - | - |
|  | **5** | u.d.l. | u.d.l. | u.d.l. | - | - | - | - |
|  | **6** | u.d.l. | u.d.l. | u.d.l. | - | - | - | - |
|  | **av.** | 2 | 10.00 | 40.00 | 1.30E-04 | 5.20E-04 | 1.02 | 4.07 |
| 10 | **1** | 7.000 | 35.00 | 140.00 | 4.55E-04 | 1.82E-03 | 3.56 | 14.24 |
|  | **2** | 7.000 | 35.00 | 140.00 | 4.55E-04 | 1.82E-03 | 3.56 | 14.24 |
|  | **3** | 8.000 | 40.00 | 160.00 | 5.20E-04 | 2.08E-03 | 4.07 | 16.28 |
|  | **4** | 1.000 | 5.00 | 20.00 | 6.50E-05 | 2.60E-04 | 0.51 | 2.03 |
|  | **5** | 1.000 | 5.00 | 20.00 | 6.50E-05 | 2.60E-04 | 0.51 | 2.03 |
|  | **6** | 1.000 | 5.00 | 20.00 | 6.50E-05 | 2.60E-04 | 0.51 | 2.03 |
|  | **av.** | 4 | 20.83 | 83.33 | 2.71E-04 | 1.08E-03 | 2.12 | 8.48 |
| 11 | **1** | 2.000 | 10.00 | 40.00 | 1.30E-04 | 5.20E-04 | 1.02 | 4.07 |
|  | **2** | u.d.l. | u.d.l. | u.d.l. | - | - | - | - |
|  | **3** | u.d.l. | u.d.l. | u.d.l. | - | - | - | - |
|  | **4** | 2.000 | 10.00 | 40.00 | 1.30E-04 | 5.20E-04 | 1.02 | 4.07 |
|  | **5** | 1.000 | 5.00 | 20.00 | 6.50E-05 | 2.60E-04 | 0.51 | 2.03 |
|  | **6** | u.d.l. | u.d.l. | u.d.l. | - | - | - | - |
|  | **av.** | 1 | 4.17 | 16.67 | 5.42E-05 | 2.17E-04 | 0.42 | 1.70 |
| 12 | **1** | 3.000 | 15.00 | 60.00 | 1.95E-04 | 7.80E-04 | 1.53 | 6.10 |
|  | **2** | 4.000 | 20.00 | 80.00 | 2.60E-04 | 1.04E-03 | 2.03 | 8.14 |
|  | **3** | 10.000 | 50.00 | 200.00 | 6.50E-04 | 2.60E-03 | 5.09 | 20.35 |
|  | **4** | 2.000 | 10.00 | 40.00 | 1.30E-04 | 5.20E-04 | 1.02 | 4.07 |
|  | **5** | 1.000 | 5.00 | 20.00 | 6.50E-05 | 2.60E-04 | 0.51 | 2.03 |
|  | **6** | u.d.l. | u.d.l. | u.d.l. | - | - | - | - |
|  | **av.** | 3 | 16.67 | 66.67 | 2.17E-04 | 8.67E-04 | 1.70 | 6.78 |
| 13 | **1** | 3.000 | 15.00 | 60.00 | 1.95E-04 | 7.80E-04 | 1.53 | 6.10 |
|  | **2** | 4.000 | 20.00 | 80.00 | 2.60E-04 | 1.04E-03 | 2.03 | 8.14 |
|  | **3** | u.d.l. | u.d.l. | u.d.l. | - | - | - | - |
|  | **4** | 2.000 | 10.00 | 40.00 | 1.30E-04 | 5.20E-04 | 1.02 | 4.07 |
|  | **5** | 1.000 | 5.00 | 20.00 | 6.50E-05 | 2.60E-04 | 0.51 | 2.03 |
|  | **6** | u.d.l. | u.d.l. | u.d.l. | - | - | - | - |
|  | **av.** | 2 | 8.33 | 33.33 | 1.08E-04 | 4.33E-04 | 0.85 | 3.39 |
| 14 | **1** | 3.000 | 15.00 | 60.00 | 1.95E-04 | 7.80E-04 | 1.53 | 6.10 |
|  | **2** | 1.000 | 5.00 | 20.00 | 6.50E-05 | 2.60E-04 | 0.51 | 2.03 |
|  | **3** | u.d.l. | u.d.l. | u.d.l. | - | - | - | - |
|  | **4** | 5.000 | 25.00 | 100.00 | 3.25E-04 | 1.30E-03 | 2.54 | 10.17 |
|  | **5** | 1.000 | 5.00 | 20.00 | 6.50E-05 | 2.60E-04 | 0.51 | 2.03 |
|  | **6** | 1.000 | 5.00 | 20.00 | 6.50E-05 | 2.60E-04 | 0.51 | 2.03 |
|  | **av.** | 2 | 9.17 | 36.67 | 1.19E-04 | 4.77E-04 | 0.93 | 3.73 |
| 15 | **1** | 1.000 | 5.00 | 20.00 | 6.50E-05 | 2.60E-04 | 0.51 | 2.03 |
|  | **2** | 2.000 | 10.00 | 40.00 | 1.30E-04 | 5.20E-04 | 1.02 | 4.07 |
|  | **3** | 2.000 | 10.00 | 40.00 | 1.30E-04 | 5.20E-04 | 1.02 | 4.07 |
|  | **4** | 1.000 | 5.00 | 20.00 | 6.50E-05 | 2.60E-04 | 0.51 | 2.03 |
|  | **5** | 1.000 | 5.00 | 20.00 | 6.50E-05 | 2.60E-04 | 0.51 | 2.03 |
|  | **6** | 1.000 | 5.00 | 20.00 | 6.50E-05 | 2.60E-04 | 0.51 | 2.03 |
|  | **av.** | 1 | 6.67 | 26.67 | 8.67E-05 | 3.47E-04 | 0.68 | 2.71 |
| 16 | **1** | 1.000 | 5.00 | 20.00 | 6.50E-05 | 2.60E-04 | 0.51 | 2.03 |
|  | **2** | 1.000 | 5.00 | 20.00 | 6.50E-05 | 2.60E-04 | 0.51 | 2.03 |
|  | **3** | 1.000 | 5.00 | 20.00 | 6.50E-05 | 2.60E-04 | 0.51 | 2.03 |
|  | **4** | 2.000 | 10.00 | 40.00 | 1.30E-04 | 5.20E-04 | 1.02 | 4.07 |
|  | **5** | 2.000 | 10.00 | 40.00 | 1.30E-04 | 5.20E-04 | 1.02 | 4.07 |
|  | **6** | 10.000 | 50.00 | 200.00 | 6.50E-04 | 2.60E-03 | 5.09 | 20.35 |
|  | **av.** | 3 | 14.17 | 56.67 | 1.84E-04 | 7.37E-04 | 1.44 | 5.76 |
| 17 | **1** | 4.000 | 20.00 | 80.00 | 2.60E-04 | 1.04E-03 | 2.03 | 8.14 |
|  | **2** | 3.000 | 15.00 | 60.00 | 1.95E-04 | 7.80E-04 | 1.53 | 6.10 |
|  | **3** | 1.000 | 5.00 | 20.00 | 6.50E-05 | 2.60E-04 | 0.51 | 2.03 |
|  | **4** | 4.000 | 20.00 | 80.00 | 2.60E-04 | 1.04E-03 | 2.03 | 8.14 |
|  | **5** | 1.000 | 5.00 | 20.00 | 6.50E-05 | 2.60E-04 | 0.51 | 2.03 |
|  | **6** | 1.000 | 5.00 | 20.00 | 6.50E-05 | 2.60E-04 | 0.51 | 2.03 |
|  | **av.** | 2 | 11.67 | 46.67 | 1.52E-04 | 6.07E-04 | 1.19 | 4.75 |
| 18 | **1** | 12.000 | 60.00 | 240.00 | 7.80E-04 | 3.12E-03 | 6.10 | 24.42 |
|  | **2** | 13.000 | 65.00 | 260.00 | 8.45E-04 | 3.38E-03 | 6.61 | 26.45 |
|  | **3** | 14.000 | 70.00 | 280.00 | 9.10E-04 | 3.64E-03 | 7.12 | 28.48 |
|  | **4** | 1.000 | 5.00 | 20.00 | 6.50E-05 | 2.60E-04 | 0.51 | 2.03 |
|  | **5** | 1.000 | 5.00 | 20.00 | 6.50E-05 | 2.60E-04 | 0.51 | 2.03 |
|  | **6** | 1.000 | 5.00 | 20.00 | 6.50E-05 | 2.60E-04 | 0.51 | 2.03 |
|  | **av.** | 7 | 35.00 | 140.00 | 4.55E-04 | 1.82E-03 | 3.56 | 14.24 |
| 19 | **1** | 8.000 | 40.00 | 160.00 | 5.20E-04 | 2.08E-03 | 4.07 | 16.28 |
|  | **2** | 15.000 | 75.00 | 300.00 | 9.75E-04 | 3.90E-03 | 7.63 | 30.52 |
|  | **3** | 15.000 | 75.00 | 300.00 | 9.75E-04 | 3.90E-03 | 7.63 | 30.52 |
|  | **4** | 5.000 | 25.00 | 100.00 | 3.25E-04 | 1.30E-03 | 2.54 | 10.17 |
|  | **5** | 11.000 | 55.00 | 220.00 | 7.15E-04 | 2.86E-03 | 5.60 | 22.38 |
|  | **6** | 11.000 | 55.00 | 220.00 | 7.15E-04 | 2.86E-03 | 5.60 | 22.38 |
|  | **av.** | 11 | 54.17 | 216.67 | 7.04E-04 | 2.82E-03 | 5.51 | 22.04 |
| 20 | **1** | 20.000 | 100.00 | 400.00 | 1.30E-03 | 5.20E-03 | 10.17 | 40.69 |
|  | **2** | 20.000 | 100.00 | 400.00 | 1.30E-03 | 5.20E-03 | 10.17 | 40.69 |
|  | **3** | 20.000 | 100.00 | 400.00 | 1.30E-03 | 5.20E-03 | 10.17 | 40.69 |
|  | **4** | 15.000 | 75.00 | 300.00 | 9.75E-04 | 3.90E-03 | 7.63 | 30.52 |
|  | **5** | 1.000 | 5.00 | 20.00 | 6.50E-05 | 2.60E-04 | 0.51 | 2.03 |
|  | **6** | 1.000 | 5.00 | 20.00 | 6.50E-05 | 2.60E-04 | 0.51 | 2.03 |
|  | **av.** | 13 | 64.17 | 256.67 | 8.34E-04 | 3.34E-03 | 6.53 | 26.11 |
| 21 | **1** | 13.333 | 66.67 | 266.67 | 8.67E-04 | 3.47E-03 | 6.78 | 27.13 |
|  | **2** | 16.000 | 80.00 | 320.00 | 1.04E-03 | 4.16E-03 | 8.14 | 32.55 |
|  | **3** | 16.333 | 81.67 | 326.67 | 1.06E-03 | 4.25E-03 | 8.31 | 33.23 |
|  | **4** | 7.000 | 35.00 | 140.00 | 4.55E-04 | 1.82E-03 | 3.56 | 14.24 |
|  | **5** | 4.333 | 21.67 | 86.67 | 2.82E-04 | 1.13E-03 | 2.20 | 8.82 |
|  | **6** | 4.333 | 21.67 | 86.67 | 2.82E-04 | 1.13E-03 | 2.20 | 8.82 |
|  | **av.** | 10 | 51.11 | 204.44 | 6.64E-04 | 2.66E-03 | 5.20 | 20.80 |
| 22 | **1** | 25.000 | 125.00 | 500.00 | 1.63E-03 | 6.50E-03 | 12.72 | 50.86 |
|  | **2** | 13.000 | 65.00 | 260.00 | 8.45E-04 | 3.38E-03 | 6.61 | 26.45 |
|  | **3** | 5.000 | 25.00 | 100.00 | 3.25E-04 | 1.30E-03 | 2.54 | 10.17 |
|  | **4** | 33.000 | 165.00 | 660.00 | 2.15E-03 | 8.58E-03 | 16.79 | 67.14 |
|  | **5** | 21.000 | 105.00 | 420.00 | 1.37E-03 | 5.46E-03 | 10.68 | 42.73 |
|  | **6** | 2.000 | 10.00 | 40.00 | 1.30E-04 | 5.20E-04 | 1.02 | 4.07 |
|  | **av.** | 17 | 82.50 | 330.00 | 1.07E-03 | 4.29E-03 | 8.39 | 33.57 |
| 23 | **1** | 62.333 | 311.67 | 1246.67 | 4.05E-03 | 1.62E-02 | 31.71 | 126.82 |
|  | **2** | 32.000 | 160.00 | 640.00 | 2.08E-03 | 8.32E-03 | 16.28 | 65.11 |
|  | **3** | 2.333 | 11.67 | 46.67 | 1.52E-04 | 6.07E-04 | 1.19 | 4.75 |
|  | **4** | 49.667 | 248.33 | 993.33 | 3.23E-03 | 1.29E-02 | 25.26 | 101.05 |
|  | **5** | 40.333 | 201.67 | 806.67 | 2.62E-03 | 1.05E-02 | 20.52 | 82.06 |
|  | **6** | 34.000 | 170.00 | 680.00 | 2.21E-03 | 8.84E-03 | 17.29 | 69.18 |
|  | **av.** | 37 | 183.89 | 735.56 | 2.39E-03 | 9.56E-03 | 18.71 | 74.83 |
| 24 | **1** | 33.556 | 167.78 | 671.11 | 2.18E-03 | 8.72E-03 | 17.07 | 68.27 |
|  | **2** | 20.333 | 101.67 | 406.67 | 1.32E-03 | 5.29E-03 | 10.34 | 41.37 |
|  | **3** | 7.889 | 39.44 | 157.78 | 5.13E-04 | 2.05E-03 | 4.01 | 16.05 |
|  | **4** | 29.889 | 149.44 | 597.78 | 1.94E-03 | 7.77E-03 | 15.20 | 60.81 |
|  | **5** | 21.889 | 109.44 | 437.78 | 1.42E-03 | 5.69E-03 | 11.13 | 44.53 |
|  | **6** | 13.444 | 67.22 | 268.89 | 8.74E-04 | 3.50E-03 | 6.84 | 27.35 |
|  | **av.** | 21 | 105.83 | 423.33 | 1.38E-03 | 5.50E-03 | 10.77 | 43.07 |
| 25 | **1** | 3.000 | 15.00 | 60.00 | 1.95E-04 | 7.80E-04 | 1.53 | 6.10 |
|  | **2** | 1.000 | 5.00 | 20.00 | 6.50E-05 | 2.60E-04 | 0.51 | 2.03 |
|  | **3** | u.d.l. | u.d.l. | u.d.l. | - | - | - | - |
|  | **4** | 10.000 | 50.00 | 200.00 | 6.50E-04 | 2.60E-03 | 5.09 | 20.35 |
|  | **5** | 1.000 | 5.00 | 20.00 | 6.50E-05 | 2.60E-04 | 0.51 | 2.03 |
|  | **6** | u.d.l. | u.d.l. | u.d.l. | - | - | - | - |
|  | **av.** | 3 | 12.50 | 50.00 | 1.63E-04 | 6.50E-04 | 1.27 | 5.09 |
| 26 | **1** | 110.000 | 550.00 | 2200.00 | 7.15E-03 | 2.86E-02 | 55.95 | 223.80 |
|  | **2** | 100.000 | 500.00 | 2000.00 | 6.50E-03 | 2.60E-02 | 50.86 | 203.46 |
|  | **3** | 50.000 | 250.00 | 1000.00 | 3.25E-03 | 1.30E-02 | 25.43 | 101.73 |
|  | **4** | 400.000 | 2000.00 | 8000.00 | 2.60E-02 | 1.04E-01 | 203.46 | 813.84 |
|  | **5** | 240.000 | 1200.00 | 4800.00 | 1.56E-02 | 6.24E-02 | 122.08 | 488.30 |
|  | **6** | 158.000 | 790.00 | 3160.00 | 1.03E-02 | 4.11E-02 | 80.37 | 321.46 |
|  | **av.** | 176 | 881.67 | 3526.67 | 1.15E-02 | 4.58E-02 | 89.69 | 358.77 |
| 27 | **1** | 159.000 | 795.00 | 3180.00 | 1.03E-02 | 4.13E-02 | 80.87 | 323.50 |
|  | **2** | 83.000 | 415.00 | 1660.00 | 5.40E-03 | 2.16E-02 | 42.22 | 168.87 |
|  | **3** | 2.000 | 10.00 | 40.00 | 1.30E-04 | 5.20E-04 | 1.02 | 4.07 |
|  | **4** | 113.000 | 565.00 | 2260.00 | 7.35E-03 | 2.94E-02 | 57.48 | 229.91 |
|  | **5** | 100.000 | 500.00 | 2000.00 | 6.50E-03 | 2.60E-02 | 50.86 | 203.46 |
|  | **6** | 100.000 | 500.00 | 2000.00 | 6.50E-03 | 2.60E-02 | 50.86 | 203.46 |
|  | **av.** | 93 | 464.17 | 1856.67 | 6.03E-03 | 2.41E-02 | 47.22 | 188.88 |
| 28 | **1** | 13.000 | 65.00 | 260.00 | 8.45E-04 | 3.38E-03 | 6.61 | 26.45 |
|  | **2** | 12.000 | 60.00 | 240.00 | 7.80E-04 | 3.12E-03 | 6.10 | 24.42 |
|  | **3** | 16.000 | 80.00 | 320.00 | 1.04E-03 | 4.16E-03 | 8.14 | 32.55 |
|  | **4** | 8.000 | 40.00 | 160.00 | 5.20E-04 | 2.08E-03 | 4.07 | 16.28 |
|  | **5** | 5.000 | 25.00 | 100.00 | 3.25E-04 | 1.30E-03 | 2.54 | 10.17 |
|  | **6** | u.d.l. | u.d.l. | u.d.l. | - | - | - | - |
|  | **av.** | 9 | 45.00 | 180.00 | 5.85E-04 | 2.34E-03 | 4.58 | 18.31 |
| 29 | **1** | 3.000 | 15.00 | 60.00 | 1.95E-04 | 7.80E-04 | 1.53 | 6.10 |
|  | **2** | 4.000 | 20.00 | 80.00 | 2.60E-04 | 1.04E-03 | 2.03 | 8.14 |
|  | **3** | 5.000 | 25.00 | 100.00 | 3.25E-04 | 1.30E-03 | 2.54 | 10.17 |
|  | **4** | 1.000 | 5.00 | 20.00 | 6.50E-05 | 2.60E-04 | 0.51 | 2.03 |
|  | **5** | u.d.l. | u.d.l. | u.d.l. | - | - | - | - |
|  | **6** | u.d.l. | u.d.l. | u.d.l. | - | - | - | - |
|  | **av.** | 2 | 10.83 | 43.33 | 1.41E-04 | 5.63E-04 | 1.10 | 4.41 |
| 30 | **1** | 309.000 | 1545.00 | 6180.00 | 2.01E-02 | 8.03E-02 | 157.17 | 628.69 |
|  | **2** | 51.000 | 255.00 | 1020.00 | 3.32E-03 | 1.33E-02 | 25.94 | 103.76 |
|  | **3** | 1.000 | 5.00 | 20.00 | 6.50E-05 | 2.60E-04 | 0.51 | 2.03 |
|  | **4** | 210.000 | 1050.00 | 4200.00 | 1.37E-02 | 5.46E-02 | 106.82 | 427.26 |
|  | **5** | 100.000 | 500.00 | 2000.00 | 6.50E-03 | 2.60E-02 | 50.86 | 203.46 |
|  | **6** | u.d.l. | u.d.l. | u.d.l. | - | - | - | - |
|  | **av.** | 112 | 559.17 | 2236.67 | 7.27E-03 | 2.91E-02 | 56.88 | 227.53 |
| 31 | **1** | 15.000 | 75.00 | 300.00 | 9.75E-04 | 3.90E-03 | 7.63 | 30.52 |
|  | **2** | 15.000 | 75.00 | 300.00 | 9.75E-04 | 3.90E-03 | 7.63 | 30.52 |
|  | **3** | 1.000 | 5.00 | 20.00 | 6.50E-05 | 2.60E-04 | 0.51 | 2.03 |
|  | **4** | 15.000 | 75.00 | 300.00 | 9.75E-04 | 3.90E-03 | 7.63 | 30.52 |
|  | **5** | 18.000 | 90.00 | 360.00 | 1.17E-03 | 4.68E-03 | 9.16 | 36.62 |
|  | **6** | 1.000 | 5.00 | 20.00 | 6.50E-05 | 2.60E-04 | 0.51 | 2.03 |
|  | **av.** | 11 | 54.17 | 216.67 | 7.04E-04 | 2.82E-03 | 5.51 | 22.04 |
| 32 | **1** | 3.000 | 15.00 | 60.00 | 1.95E-04 | 7.80E-04 | 1.53 | 6.10 |
|  | **2** | u.d.l. | u.d.l. | u.d.l. | - | - | - | - |
|  | **3** | u.d.l. | u.d.l. | u.d.l. | - | - | - | - |
|  | **4** | 3.000 | 15.00 | 60.00 | 1.95E-04 | 7.80E-04 | 1.53 | 6.10 |
|  | **5** | u.d.l. | u.d.l. | u.d.l. | - | - | - | - |
|  | **6** | u.d.l. | u.d.l. | u.d.l. | - | - | - | - |
| For all samples | **av.** | 1 | 5.00 | 20.00 | 6.50E-05 | 2.60E-04 | 0.51 | 2.03 |
|  | Max. | 400.000 | 2000.00 | 8000.00 | 2.60E-02 | 1.04E-01 | 203.46 | 813.84 |
|  | Av. | 21.057 | 105.29 | 421.15 | 1.37E-03 | 5.47E-03 | 10.71 | 42.84 |
|  | Me | 4.167 | 20.83 | 83.33 | 2.71E-04 | 1.08E-03 | 2.12 | 8.48 |
|  | SD | 50.576 | 252.88 | 1011.52 | 3.29E-03 | 1.31E-02 | 25.73 | 102.90 |

u.d.l. – under detection limit
